# Impact of Parathyroidectomy on Kidney Function in Adults With Primary Hyperparathyroidism

**DOI:** 10.7759/cureus.99762

**Published:** 2025-12-21

**Authors:** João Pedro Bandovas, Henrique Candeias, Mariana Mourão, Anjum Dhanani, Nuno Monteiro, Ana Crespo, Paula Tavares, Hugo Pinto Marques

**Affiliations:** 1 General Surgery, Unidade Local de Saúde de S. José - Hospital Curry Cabral, Lisbon, PRT; 2 NOVA Medical School, Universidade NOVA de Lisboa (NMS/UNL), Lisbon, PRT

**Keywords:** chronic kidney disease (ckd), elective parathyroidectomy, primary hyperparathyroidism (phpt), renal function, surgical indication

## Abstract

Introduction

Primary hyperparathyroidism (PHPT) is characterized by persistent hypercalcemia and is associated with renal complications, including nephrolithiasis and progressive decline in the estimated glomerular filtration rate (eGFR). Although parathyroidectomy (PTX) is the definitive treatment, its impact on renal function remains uncertain, particularly in patients with pre-existing renal impairment. This study aims to evaluate 12-month changes in renal function after PTX in patients with PHPT, according to baseline kidney function.

Methods

This retrospective study included 48 patients with PHPT who underwent PTX between 2017 and 2020. Patients were stratified by baseline eGFR into two groups: ≥60 mL/min/1.73 m² (Group 1) and <60 mL/min/1.73 m² (Group 2). Clinical and laboratory parameters, including serum creatinine and eGFR, were analyzed at baseline and at 12 months postoperatively.

Results

Patients were predominantly women (ratio 3.8:1), and the surgical cure rate was 95.8%. Group 2 presented higher baseline calcium and PTH levels. At 12 months, both groups showed significant reductions in calcium and PTH. Group 1 experienced a statistically significant decline in eGFR, whereas Group 2 showed a slight, non-significant improvement, suggesting stabilization of renal function.

Conclusion

PTX does not appear to improve renal function in most patients with PHPT but may prevent further deterioration in those with pre-existing renal impairment. These findings support current guideline recommendations favoring surgical intervention in PHPT patients with compromised baseline kidney function.

## Introduction

Primary hyperparathyroidism (PHPT) is a common endocrine disorder characterized by excessive parathyroid hormone secretion, leading to hypercalcemia and a spectrum of end-organ complications, including nephrolithiasis and chronic kidney disease (CKD). The relationship between PHPT and renal dysfunction is well established, with hypercalcemia and hypercalciuria contributing to a progressive decline in glomerular filtration rate (eGFR) [1,2].

Parathyroidectomy (PTX) is the only definitive treatment for PHPT, but its impact on renal function remains incompletely understood. The most recent guidelines for the management of primary hyperparathyroidism note that the use of PTX with the specific intent of preserving kidney function is supported by low-quality, limited and inconsistent evidence [3,4]. Indeed, long-term data suggest that PTX may slow the rate of eGFR decline in patients with more severe hypercalcemia or pre-existing renal impairment, particularly those with baseline eGFR <60 mL/min/1.73 m² [1,5-7]. In contrast, randomized controlled trial data indicate that PTX does not significantly alter renal outcomes in patients with mild PHPT or in older adults, suggesting that the renal benefits of surgery are limited to select subgroups [8,9].

This study aims to evaluate 12-month changes in renal function following PTX in patients with PHPT, according to baseline kidney function. We hypothesize that patients with some degree of renal impairment may benefit from surgery, in line with current guidelines.

This article was previously presented as a meeting abstract at the 61st European Renal Association (ERA) Congress on May 24, 2024.

## Materials and methods

Study population

Consecutive patients with PHPT who underwent PTX at Hospital Curry Cabral between January 2017 and December 2020 were included in this retrospective study.

PHPT was diagnosed by demonstrating persistent hypercalcemia (serum calcium (SCa) higher than 10.2 mg/dL) in the presence of inappropriately normal (>15 pg/mL) or elevated (>65 pg/mL) PTH concentrations. Exclusion criteria comprised patients without at least one-year follow-up or with missing data before and 12 months after surgery, prior nephrectomy and diagnosis of multiple endocrine neoplasia. PHPT was classified as symptomatic in patients presenting with overt symptoms related to nephrolithiasis, bone manifestations (bone or joint pain or fractures) or general symptoms attributable to PHPT (gastrointestinal or neuromuscular).

Indications for surgery were in accordance with guidelines applied at the time of the diagnosis [4]. Patients with symptomatic PHPT or asymptomatic with at least one of the criteria of age <50 years, SCa level greater than 1 mg/dL above normal, the presence of kidney stones, eGFR <60 mL/min, or bone demineralization underwent PTX after preoperative localization imaging, including cervical ultrasound and further four-dimensional CT in selected cases.

A simple adenectomy was performed for single adenomas. In patients with multi-gland involvement or when there was concomitant thyroid pathology or a negative localization study, the approach of a full neck exploration was adopted. During surgery, intact PTH (iPTH) was monitored and a decrease of more than 50% from pre-operative value was considered predictive of biochemical cure.

Study parameters

Sociodemographic variables including age, sex, body mass index, comorbid conditions (history of diabetes, hypertension and angiotensin-converting enzyme inhibitors/angiotensin receptor blockers (ACEi/ARB) use; history of nephrolithiasis or bone fracture/involvement; associated symptoms) and laboratory parameters (SCa, albumin, phosphorus, PTH, vitamin D, creatinine, and eGFR) were collected from the last data before PTX and at 12 months after surgery.

Patients who were receiving blood pressure-lowering treatment, regardless of the blood pressure values recorded, were considered hypertensive. Bone mineral density (BMD) was measured by dual-energy X-ray absorptiometry (DEXA) in the lumbar spine (L2-L4) and femur. A patient was considered to have bone involvement when DEXA showed a decrease in bone mineral density (T score <-2.5 SD in the spine or femur).

Serum total calcium, phosphate and creatine were analyzed by a standard autoanalyzer using colorimetric and enzymatic methods. PTH was obtained using electrochemiluminescent immunoassay. The eGFR was calculated using the Chronic Kidney Disease Epidemiology Collaboration (CKD-EPI) formula. 

All evaluations were obtained during a single visit at the time of the diagnosis and subsequently at the follow-up. Patients gave informed consent to these investigations as part of their normal care both at diagnosis and follow-up.

Patients were dichotomized into two groups according to their baseline eGFR: mild CKD or better, defined as eGFR³ 60 mL/min (group 1), and moderate CKD or worse, eGFR <60 mL/min (group 2). The main outcome was the raw change of kidney function (serum creatinine and eGFR) between baseline and at 12 postoperative months in the two groups.

Statistical analysis

Comparisons between groups (unpaired series) or between baseline and 12-month postoperative (paired series) were performed using Student’s t-test for continuous variables and Fischer exact test for categorical variables. The results were considered statistically significant for p<0.05.

Statistical analyses were performed using the software program IBM SPSS Statistics, version 23 (IBM Corp, Armonk, NY).

## Results

Ninety patients underwent PTX between January 2017 and December 2020. Of these, 48 patients were included in the analysis. The mean age of the patients was 67.1±6.36 years and the female-to-male ratio was 3.8:1. Hypertension was more common than diabetes mellitus (75% vs. 33%, respectively), and both conditions occurred simultaneously in a substantial proportion of patients (n=15, 31%). Twenty-three patients (47.9%) had documented nephrolithiasis. Although pathological fractures were uncommon (n=2, 4.1%), bone involvement was highly prevalent (37 patients, 77.1%). In 54.2% of patients, the indication for PTX was symptomatic of PHPT.

Thirty-five patients (72.9%) presented with a preoperative eGFR≥60 mL/min and were assigned to Group 1, whereas 13 patients (27.1%) had a preoperative eGFR<60 mL/min and were assigned to Group 2. Patients in the two groups did not differ with regard to age, gender, presence of PHPT symptoms and comorbidities. Table 1 summarizes the patients’ baseline characteristics of both groups.

**Table 1:** Baseline demographics and clinical characteristics Abbreviations: eGFR, Estimated glomerular filtration rate; BMD, bone mineral density; n.a., not applicable. Continuous variables are presented as mean±SD. Categorical variables are presented as number (percentage). Comparisons between groups were performed using Student’s t-test for the continuous variables and Fischer exact test for the categorical variables. The results were considered statistically significant for p<0.05.

	eGFR≥60 mL/m^2 ^(n = 35)	eGFR<60 mL/m^2^ (n = 13)	t (df); *p* value
Age (years)	66.8±11.7	72.6±9.1	-1.61 (46); 0.115
Female	27 (77.1%)	11 (84.6%)	n.a.; 0.571
Diabetes	11 (31.4%)	5 (38.5%)	n.a.; 0.646
Hypertension	24 (68.6%)	12 (92.3%)	n.a.; 0.091
Symptoms	18 (51.4%)	8 (61.5%)	n.a.; 0.532
Kidney Stones	19 (57.6%)	4 (30.8%)	n.a.; 0.102
BMD (T-score)	-2.61 ± 1.00	-2.68 ± 0.97	0.19 (35); 0.854

Mean sCa and PTH levels were significantly higher in Group 2 (12.09±1.20 vs. 11.19±0.55 mg/dL and 384.9±244.0 vs. 229.7±145.7 pg/mL). At the 12-month follow-up, 46 of 48 patients (95.8%) were cured, and the remaining patients achieved cure after reoperation. Serum calcium and PTH levels decreased significantly in all patients, and the physiologically expected increases in serum phosphorus and vitamin D were observed; however, mean PTH levels remained slightly elevated in Group 2 at the 12-month follow-up (Table 2).

**Table 2:** Laboratory parameters comparison, before surgery and at 12 months. Abbreviations: eGFR, Estimated glomerular filtration rate; PTH, parathyroid hormone; pre-op, preoperative; pos-op, postoperative. Continuous variables are presented as mean±SD. Comparisons between baseline and 12-month postoperative were performed using Student’s t-test on paired data. The results were considered statistically significant for p<0.05.

	eGFR≥60 mL/m^2 ^ (n = 35)			eGFR<60 mL/m^2 ^ (n = 13)		
	Pre-Op	Post-Op	t (df); p value	Pre-Op	Post-Op	t (df); p value
PTH (pg/mL)	229.7±145.74	70.0±30.05	6.88 (34); 0.000	384.9±244.0	106.5±67.05	3.96 (11); 0.002
Calcium (mg/dL)	11.19±0.55	9.33±0.41	17.09 (34); 0.000	12.09±1.20	9.28±0.51	7.33 (12); 0.000
Phosphorus (mg/dL)	2.61±1.13	3.21±0.61	-2.92 (32); 0.006	2.55±0.56	3.36±0.44	-4.20 (11); 0.001
Vitamin D (ng/mL)	16.26±6.51	30.64±11.09	-3.92 (13); 0.002	22.58±17.99	28.10±19.94	-1.93 (4); 0.127
Creatinine (mg/dL)	0.78±0.12	0.86±0.26	-2.12 (34); 0.042	1.35±0.32	1.42±0.46	-0.48 (12); 0.638
eGFR (mL/m2)	88.03±12.68	82.20±17.75	2.64 (34); 0.012	44.62±9.47	45.08±17.54	-0.09 (12); 0.931

**Figure 1:**
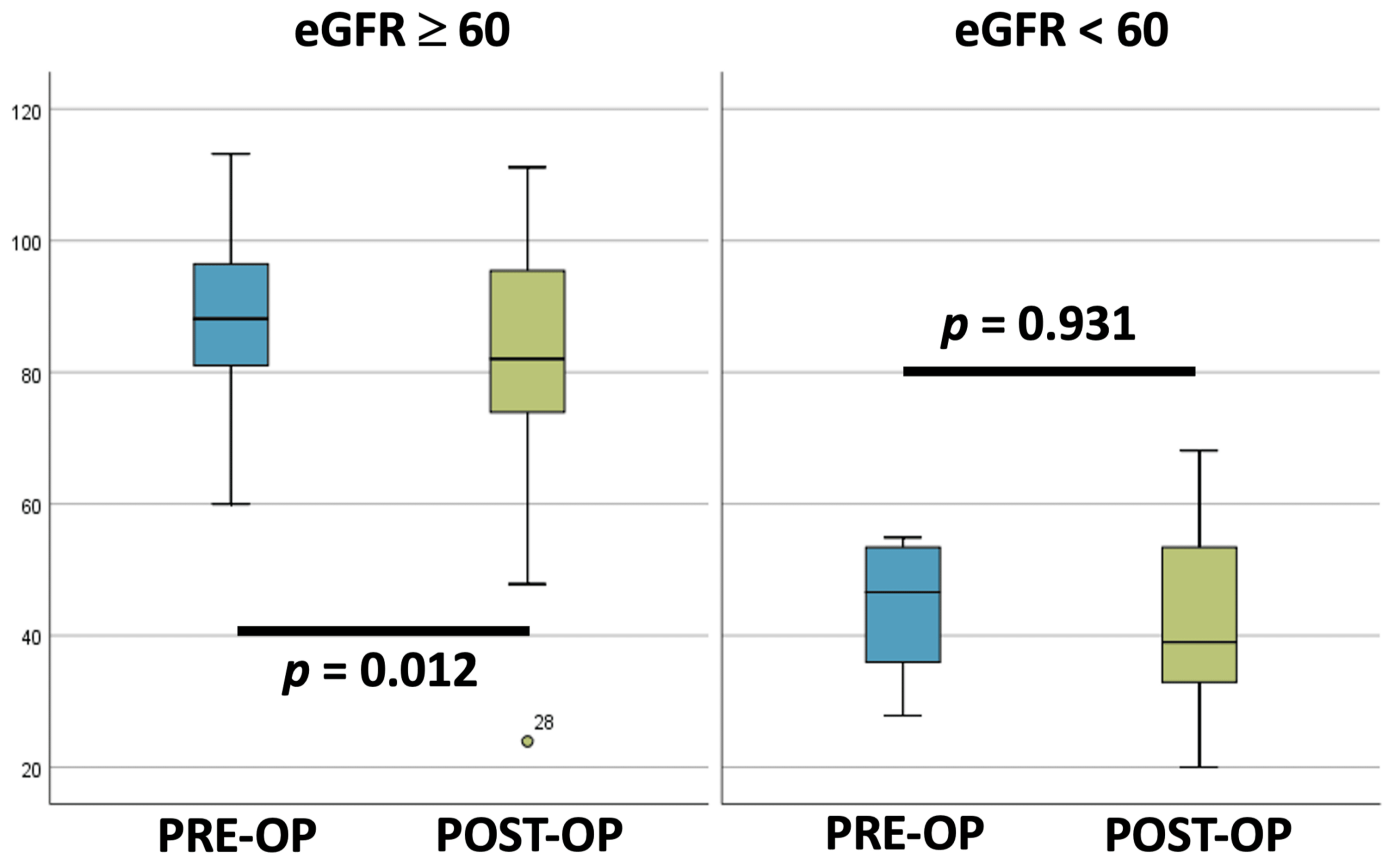
eGFR variation after parathyroidectomy by subgroup. Change of kidney function parameters one year after parathyroidectomy, according to eGFR. Comparisons between baseline and 12-month postoperative were performed using Student’s t-test on paired data (t-values presented on Table 2). The results were considered statistically significant for p<0.05. eGFR: Estimated glomerular filtration rate.

Regarding the impact of PTX on kidney function, Group 1 showed a statistically significant decrease in eGFR at 12 months post-PTX, whereas patients with pre-existing impaired kidney function (Group 2) appeared to experience a slight improvement, although this change did not reach statistical significance (Figure 1).

## Discussion

Parathyroidectomy is the definitive treatment and provides a complete cure for PHPT. In our cohort, the cure rate is over 95% after primary surgery. Despite higher blood calcium levels in the CKD group, calcium returned to normal in all patients, whereas phosphate levels increased. PTH, which was also higher at baseline, remained mildly elevated in CKD group. As noted by Dal Osto et al., renal dysfunction is a predictive factor for persistent PTH after surgery, though this does not necessarily indicate PHPT persistence or recurrence [10].

In recent decades, the clinical presentation of PHPT has shifted markedly, with approximately 80%-90% of cases in developed countries now being identified as asymptomatic at diagnosis [11]. CKD is also recognized as a common manifestation in PHPT, reported in 13%-19% of this population [12]. In contrast, in our cohort, only 45.9% of patients were asymptomatic and a higher proportion (27.1%) had CKD, findings that likely reflects selection bias given that our study population consisted exclusively of individuals meeting criteria for PTX.

In the present study, PTX prevented further deterioration on renal function in PHPT patients with coexisting renal impairment, a finding consistent with results from multiple prospective and retrospective studies. Frey et al. found that nearly half of such patients improved their chronic kidney disease stage after PTX, and only a small minority worsened [5]. Tassone et al. similarly reported that PTX halted renal function decline in patients with impaired baseline eGFR, supporting surgical intervention in this group [6]. Zhu et al. demonstrated that PTX mitigated accelerated eGFR decline in patients with severe hypercalcemia (>2.87 mmol/L) [1]. PTX restores calcium and parathyroid hormone homeostasis, thereby mitigating the factors that contribute to renal injury: hypercalcemia-induced hypercalciuria leads to osmotic diuresis and dehydration, renal stones and nephrocalcinosis that also contribute to tubulointerstitial inflammation; and chronic-elevated PTH has been found to promote atherosclerosis in glomerular endothelium and inflammation in proximal tubular cells [12]. However, conflicting results exist. Seib et al. found no significant difference in long-term risk of sustained eGFR decline between PTX and nonoperative management in a large cohort, except for a possible benefit in patients younger than 60 years [13]. The Cochrane review concluded that PTX may have little or no effect on hospitalizations for renal impairment, and the evidence for benefit in bone mineral density, cardiovascular outcomes, or quality of life is very uncertain [14].

In the other group of patients without baseline CKD, kidney function decreased slightly but significantly after PTX, consistent with previously cited reports [6,15,16]. The mechanisms involved in eGFR deterioration in patients with normal kidney function need to be clarified but may involve the reversal of the physiological adaptation to chronic hypercalcemia after parathyroidectomy. The sudden decrease in PTH and calcium after surgery leads to hemodynamic changes, including reduced renal vasodilation and decreased glomerular hyperfiltration, which had previously compensated for the chronic hypercalcemic state. This can result in an acute drop in eGFR, and in some cases, a small but persistent reduction in renal function may occur postoperatively.

The limitations of our study include its single-center design, which may restrict the generalisability of our findings to other populations and institutional settings. The relatively small sample size and the retrospective observational design also preclude establishing a causal relationship between changes in renal function and PTX. In addition, we did not collect early postoperative eGFR measurements. Previous studies have shown that PTX may lead to transient renal impairment in the immediate postoperative period, potentially influencing subsequent eGFR trajectories [15,16]. Longer-term effects beyond our follow-up period may exist but could not be captured in this analysis. Finally, we did not compare the effectiveness of PTX versus nonoperative management in preventing deterioration of renal function. The observed decrease in eGFR among patients without CKD might actually reflect a positive outcome when contrasted with the expected natural progression, since according to Liang et al., although PTX does not reverse renal function decline, it may slow its progression relative to the preoperative course [7].

## Conclusions

Parathyroidectomy is a highly effective treatment for primary hyperparathyroidism, achieving biochemical cure in the vast majority of patients. Although PTX does not generally improve renal function in most PHPT patients, it may help prevent further decline, particularly in those with pre-existing renal impairment. These findings support current guideline recommendations to consider PTX in PHPT patients with compromised baseline kidney function. Further studies with larger cohorts and longer follow-up are warranted to better define the long-term renal benefits of surgery and to clarify the mechanisms underlying the decline in renal function observed in patients with preserved baseline kidney function.
